# CS21 positive multidrug-resistant ETEC clinical isolates from children with diarrhea are associated with self-aggregation, and adherence

**DOI:** 10.3389/fmicb.2014.00709

**Published:** 2014-12-17

**Authors:** Ariadnna Cruz-Córdova, Karina Espinosa-Mazariego, Sara A. Ochoa, Zeus Saldaña, Gerardo E. Rodea, Vicenta Cázares-Domínguez, Viridiana Rodríguez-Ramírez, Carlos A. Eslava-Campos, Armando Navarro-Ocaña, José Arrellano-Galindo, Rigoberto Hernández-Castro, Oscar G. Gómez-Duarte, Firdausi Qadri, Juan Xicohtencatl-Cortes

**Affiliations:** ^1^Unidad de Hemato-Onocología e Investigación, Laboratorio de Investigación en Bacteriología Intestinal, Hospital Infantil de México Federico GómezMexico City, Mexico; ^2^Departamento de Genética Molecular, Instituto de Fisiología Celular, Universidad Nacional Autónoma de MéxicoMexico City, Mexico; ^3^Unidad de Hemato-Onocología e Investigación, Laboratorio de Patogenicidad Bacteriana, Hospital Infantil de México Federico GómezMexico City, Mexico; ^4^Departamento de Salud Pública, Facultad de Medicina, Universidad Nacional Autónoma de MéxicoMexico City, Mexico; ^5^Laboratorio de Infectología, Departamento de Infectología, Hospital Infantil de México Federico GómezMexico City, Mexico; ^6^Departamento de Ecología de Agentes Patógenos, Hospital General “Dr. Manuel Gea González,”Mexico City, Mexico; ^7^Department of Pediatrics, Division of Pediatric Infectious Diseases, Vanderbilt University School of MedicineNashville, TN, USA; ^8^Centre for Vaccine Sciences, International Centre for Diarrhoeal Disease ResearchDhaka, Bangladesh

**Keywords:** ETEC, CS21, multidrug-resistance, biofilm, self-aggregation and adherence

## Abstract

**Background:** Enterotoxigenic *Escherichia coli* (ETEC) colonize the human intestinal mucosa using pili and non-pili colonization factors (CFs). CS21 (also designated Longus) is one of the most prevalent CFs encoded by a 14 kb *lng* DNA cluster located in a virulence plasmid of ETEC; yet limited information is available on the prevalence of CS21 positive ETEC isolates in different countries. The aim of this study was to evaluate the prevalence of CS21 among ETEC clinical isolates from Mexican and Bangladeshi children under 5 years old with diarrhea and to determine the phenotypic and genotypic features of these isolates.

**Methods:** ETEC clinical isolates positive to *lngA* gene were characterized by genotype, multidrug-resistance, self-aggregation, biofilm formation, and adherence to HT-29 cell line.

**Results:** A collection of 303 *E. coli* clinical isolates were analyzed, the 81.51% (247/303) were identified as ETEC, 30.76% (76/247) were *st*^+^/*lt*^+^, and 25.10% (62/247) were positive for the *lngA* gene. Among the *lngA*^+^ ETECs identified, 50% of isolates (31/62) were positive for LngA protein. The most frequent serotype was O128ac:H12 found in 19.35% (12/62) of *lngA*^+^ ETEC studied. Multidrug-resistance (MDR) *lngA*^+^ ETEC isolates was identified in 65% (39/60), self-aggregation in 48.38% (30/62), and biofilm formation in 83.87% (52/62). ETEC *lngA*^+^ isolates were able to adhere to HT-29 cells at different levels. Two *lngA* isogenic mutants were constructed in the ETEC E9034A and ETEC73332 clinical isolate, showing a 77% and 98% reduction in adherence, respectively with respect to the wild type.

**Conclusion:** ETEC isolates that have the *lngA* gene showed features associated with self-aggregation, and adherence to HT-29 cells, important characteristics in the human gut colonization process and pathogenesis.

## Introduction

Bacterial adherence is the first and most important step in bacterial infection. Diverse pathogenic mechanisms allow the bacteria to adhere to host tissues and aggregate at the infection site with the subsequent colonization and dissemination to other anatomical sites within the host (Nataro and Kaper, 1998; Craig et al., 2004). Enterotoxigenic *Escherichia coli* (ETEC), a leading traveler's diarrhea etiologic agent, also cause 300,000–500,000 deaths annually in children under the age of 5 years living in developing countries (WHO, 2006). Profuse watery diarrhea is the disease's hallmark, and similarly to cholera, it is mediated by the secretion of a cholera-like heat-labile (LT) and/or heat-stable toxins (ST) (Nataro and Kaper, 1998). These enterotoxins are believed to be encoded by large virulence plasmids and co-regulated with pili and non-pili surface proteins (Brinton, 1965; Beachey, 1981). Colonization of the small bowel mucosa by ETEC isolates is mediated by a broad variety of fimbrial and non-fimbrial surface structures called colonization factors (CFs). CFs includes colonization factor antigens (CFAs), coli surface antigens (CSs), and putative colonization factors (PCFs) (Gaastra and Svennerholm, 1996; Nataro and Kaper, 1998). More than 25 CFs have been described in human ETEC isolates (Qadri et al., 2005) and they are strongly associated with ETEC infection. Evidence of the role of CFs in pathogenesis include a study reporting that pili-less ETECs rarely induce diarrhea (Ahren and Svennerholm, 1985). CFs are highly prevalent in different geographic regions of the world and recognized by specific receptors not yet described (Gaastra and Svennerholm, 1996; Nataro and Kaper, 1998; Isidean et al., 2011). Epidemiological studies have shown that CFs produced by ETEC have immunogenic properties, and also have a protective immunity, which can be achieved through multiple infections by the host (Ahren and Svennerholm, 1985; Nataro and Kaper, 1998).

Human ETEC isolates express CS21 (Longus), a type IV pilus (T4P), with >20 μm in length. CS21 is expressed at 37°C when grown on blood agar plates and Pleuropneumoniae-Like Organisms (PPLO) agar (Giron et al., 1994; Mazariego-Espinosa et al., 2010). CS21 is composed of a single repeating structural protein of 22-kDa called LngA. The *lngA* gene, encoding the LngA major subunit, is contained in a large virulence plasmid of 90 kb (Giron et al., 1994). The N-terminal amino acid sequence of the LngA protein shares homology with the CofA (CFA/III) pilin subunit ETEC, TcpA of *V*. *cholerae*, and BfpA of enteropathogenic *E. coli* (Taylor et al., 1987; Girón et al., 1991, 1997; Giron et al., 1994). The gene cluster organization and amino acid sequence identity, suggest that these genetically diverse T4P may have a common ancestor (Taniguchi et al., 2001).

CS21 plays an important role in the adherence to intestinal epithelial cells (HT-29, Caco-2, and T84 cells) and display twitching motility activity; likewise, it was shown that CS21 assembly is also influenced by nutritional growth conditions that phenotypically affect the expression of LngA (Mazariego-Espinosa et al., 2010). In addition, CS21 is associated with bacterial self-aggregation, protection against environmental stress, biofilm formation, and adherence to primary intestinal epithelial cells and recently (Guevara et al., 2013) demonstrated the role of CS21 in the pathogenesis of ETEC *in vivo* using a neonatal mice challenge infection model (Clavijo et al., 2010; Guevara et al., 2013). Human-ETEC isolates encoding CFAs isolates from different geographic regions (Argentina, Bangladesh, Chile, Brazil, Egypt, and Mexico), have shown a wide distribution of the *lngA* gene (Girón et al., 1995a; Gutierrez-Cazarez et al., 2000; Pichel et al., 2002). In addition to CS21, ETEC has another T4P called CFA/III for which *cofA* is the major subunit, both are paralogous in nature and belong to evolutionarily distinct types of fimbriae (Gomez-Duarte et al., 2007); however, CFA/III has lower prevalence than CS21 (Honda et al., 1984, 1985; Isidean et al., 2011). The aim of this work was to genotypically and phenotypically characterize a collection of clinical *lngA*^+^ ETEC isolates through PCR, resistance profiles, self-aggregation properties, biofilm formation, and adherence to HT-29 cells to further understand if these attributes are related to the pathogenesis of CS21 positive clinical isolates from children with diarrhea.

## Materials and methods

### Bacterial isolates

Previously, clinical isolates identified as ETEC by serotyping were obtained from an *E. coli* culture collection repository at the Facultad de Medicina from Universidad Nacional Autónoma de México (UNAM) for the last three decades. All clinical ETEC were isolated from stool samples of children under 5 years old with diarrhea from México and Bangladesh (Table S1). Isolates were kept at −70°C in Brain Heart Infusion broth (BHI, Difco, New Jersey, USA) with 15% glycerol, until their use. The ETEC isolates were cultured on 5% sheep blood agar plates (BBL, Franklin Lakes, NJ) at 37°C and on MacConkey agar (Difco, New Jersey, USA).

### Identification of virulence genes by PCR

Genomic DNA was purified using a commercial Wizard kit (Promega, USA) from a bacterial culture grown overnight in Luria Bertani broth (LB, Difco, New Jersey, USA) at 37°C. PCR assays were performed using a commercial PCR kit (Promega, USA) with the following reaction mixtures: 5 μl of reaction buffer [MgCl_2_ (50 mM); *Taq* polymerase (0.l U), and dNTPs (2 mM)], 0.5 μl of the forward primer (10 μM), 0.5 μl of the reverse primer (10 μM), 3 μl of water, and 1 μl of DNA (100 ng) from each isolate. PCR reactions were conducted in a thermal cycler (Applied Biosystems GeneAmp PCR system 9700, NewYork, USA), with the specific melting temperature for each primer (Table 1). PCR products were separated on a 1.5% agarose gel in TAE (Tris-Acetate-EDTA) at 100 v, stained with ethidium bromide (5 μg/ml), and visualized by UV transilluminator (Bio-Imaging Systems, AccesoLab, México, D.F., México). To identify the *st*, *lt*, *lngA*, and *cs3* and *cfaI* genes in clinical isolates, the genomic DNAs of ETEC E9034A (O8:H9, *st*^+^, *lt*^+^, *lngA*^+^, and *cs3*^+^ genes) and ETEC H10467 (*cfaI*^+^ gene) were used as positive controls; whereas, the genomic DNA of *E. coli* HB101 was used as negative control. Based on PCR results, ETEC isolates positive for the *lngA* gene were selected for further characterization.

**Table 1:** **Primer sequences, amplicon size, and melting temperatures used in this study**.

**Gene**	**Primer**	**Sequence 5′-3′**	**Size (bp)**	**Tm (°C)**	**References**
*lngA*	F (J5)	ATG AGC CTG CTG GAA GTT ATC ATT G	608	62	Mazariego-Espinosa et al., 2010
	R (J6)	TTA ACG GCT ACC TAA AGT AAT TGA GTT			
*St*	F	ATT TTT CTT TCT GTA TTG TCT T	190	50	Perez et al., 2010
	R	CAC CCG GTA CAA GCA GGA TT			
*Lt*	F	GGC CAC AGA TTA TAC CGT GC	450	50	Levy, 2002
	R	CGG TCT CTA TAT TCC CTG TT			
*cfaI*	F	GGT GCA ATG GCT CTG ACC ACA	478	55.7	This study
	R	AGT AGT ATC TCT TGT AAT GAC			
*cooA (csI)*	F	GTC CAC ACC ATC AAC ACC GTT	320	62	This study
	R	ATT ATC CTG ACT AAG TCA ACG			
*cs3*	F	GGG CCC ACT CTA ACC AAA GAA	401	55.7	This study
	R	TTT AGT TTC AGG TAA TTA CCG			
*cofA* (*cfaIII*)	F	ATC CTT TCG GTT TAT AACAGA ACG G	713	45	This study
	R	CGG CTC GCC AAA GTA ATA GAG			

st: gene coding for a heat-stable enterotoxin; lt: gene coding for a heat-labile enterotoxin; lngA: gene coding for the major subunit of longus; cfaI: gene coding for the colonization factor I; cooA: gene coding for the major subunit of CSI; cs3: gene coding for the CS3; cofA: gene coding for the major subunit of CFA/III.

### Immunoblot

Whole cell extracts obtained from ETEC isolates positive for *lngA* were harvested from PPLO (Difco, New Jersey, USA), adjusted to the same concentration (OD_600_ = 1.0), boiled for 5 min with sample buffer and subjected to sodium dodecyl-sulfate polyacrylamide gel electrophoresis (SDS-PAGE) (Laemmli, 1970; Xicohtencatl-Cortes et al., 2007). Proteins separated in a 16% SDS-PAGE were electroblotted onto nitrocellulose membranes (Bio-Rad, Hercules, CA, USA) and then incubated with rabbit anti-CS21 antibodies (1:3,000 dilution) followed by incubation with a secondary goat anti-rabbit immunoglobulin G (IgG) antibody conjugated to alkaline phosphatase (diluted 1:30,000). Blots were developed with BCIP/NTM alkaline phosphatase antibody detection reagent substrate (Millipore, Darmstadt, Germany).

### Antibiotic susceptibility

Antibiotic susceptibility test was determined for ETEC isolates positive for the *lngA* gene by the Kirby-Bauer disk-diffusion method as recommended by the Clinical and Laboratory Standards Institute 2013 (CLSI-2013). Five colonies from each isolate were grown in Mueller Hinton (MH) broth (Becton Dickinson, Maryland, USA) at 37°C with constant shacking for 2–5 h until reaching an optical density at 600 nm (OD_600 nm_) equivalent to 0.5 on the McFarland scale. MH agar plates were massively seeded with bacterial suspension using a sterile swab. Discs with the appropriate antibiotics were placed on the inoculated plates and incubated at 37°C for 18–24 h. For susceptibility testing a total of 12 antibiotic categories were used: cephalosporins I/II: 30 μg cephalothin (Becton Dickinson, Maryland, USA) and 30 μg cefaclor (Becton Dickinson, Maryland, USA); carbapenems: 10 μg meropenem (Oxoid Sunnyvale, California, USA) and 10 μg imipenem (Becton Dickinson, Maryland, USA); quinolones: 30 μg nalidixic acid (Becton Dickinson, Maryland, USA); penicillins: 10 μg ampicillin (Becton Dickinson, Maryland, USA); β-Lactam/β-lactamase inhibitor combination: 20/10 μg amoxicillin-clavulanate (Becton Dickinson, Maryland, USA), 100/10 μg piperacillin-tazobactam (Oxoid Sunnyvale, California, USA), and 75/10 μg ticarcillin-clavulanate (Oxoid Sunnyvale, California, USA); tetracyclines: 30 μg tetracycline (Becton Dickinson, Maryland, USA); folate pathway inhibitors: 1.25/23.75 μg trimethoprim-sulfamethoxazole (Becton Dickinson, Maryland, USA); aminoglycosides: 10 μg gentamicin (Becton Dickinson, Maryland, USA) and 30 μg amikacin (Becton Dickinson, Maryland, USA); phenicols: 30 μg chloramphenicol (Becton Dickinson, Maryland, USA); fluoroquinolones: 5 μg ciprofloxacin (Becton Dickinson, Maryland, USA); nitrofurans: 300 μg nitrofuratoin (Becton Dickinson, Maryland, USA); monobactams: 30 μg aztreonam (Becton Dickinson, Maryland, USA); and cephalosporin III/IV: 30 μg ceftriaxone (Becton Dickinson, Maryland, USA), 30 μg ceftazidime (Becton Dickinson, Maryland, USA), and 30 μg cefepime (Becton Dickinson, Maryland, USA). Inhibition zones were determined and interpreted according to the recommendations of the CLSI-2013. *E. coli* ATCC (American Type Culture Collection) 25922 was used as quality control.

### Self-aggregation assay

The bacterial self-aggregation phenotype was analyzed using flat-bottom 24-well tissue culture plates (Corning, NY, USA); the assay consists of bacterial clumping during growth in liquid media. Bacteria grown overnight in Terrific broth (TB) (Amresco LLC, Ohio, USA) at 37°C were subcultured using a 1:100 dilution and incubated at 37°C in TB for 4 h. Self-aggregates were visualized directly on a bacterial suspension by an inverted light microscope (Olympus, Center Valley, PA) (Clavijo et al., 2010). Triplicates of the assays were performed at three different times. ETEC isolates: E9034A and *lngA*^−^ clinical isolate 114246 (background: O132:H25, *st*^+^, *lt*^+^, *ecpA*^+^) were used as positive and negative controls, respectively. To establish the role of *lngA* in self-aggregation, a comparison between isogenic mutants E9034A vs. E9034Δ*lngA::km* and 73332 vs. 73332Δ*lngA::km* was made.

### Biofilm formation

ETEC clinical isolates *lngA* positive were quantitatively analyzed according to the protocol described by Saldaña et al. (2009). In brief, 24-well plates containing 1 ml of PPLO were inoculated with 10 μl (1.5 × 10^8^ bacteria/ml) of bacterial suspensions and incubated at 37°C for 24 h. Biofilms on the surface of the wells were slowly washed three times with phosphate buffer saline 1x (PBS) (pH 7.4) and fixed with 2% formalin at 4°C overnight. Wells with fixed biofilms were decanted, washed three times with PBS and stained with 1 ml of 1% crystal violet for 20 min. The excess of crystal violet was removed and the plates washed with water twice. Subsequently, 1 ml of 70% methanol was added for biofilm quantification by measuring optical density at 600 nm. Assays were made in Triplicates and repeated three consecutive times. Enteroaggregative *E. coli* 042 and ETEC *lngA*^−^ clinical isolate 114246 were used as positive and negative controls, respectively. ETEC *lngA*^−^ absorbance was used to classify the biofilm formation into four categories as described by Saxena et al. (2014): non-biofilm, weakly, moderately, or strongly biofilm formers based upon the ODs of bacterial films (Saxena et al., 2014). The cut-off OD_600 nm_ for a 24 wells-plates is defined as two standard deviations above the mean OD_600 nm_ of the negative control. To establish the role of *lngA* in biofilm formation, a comparison between isogenic mutants E9034A vs. E9034Δ*lngA::km* and 73332 vs. 73332Δ*lngA::km* was performed.

### Bacterial adherence to HT-29 cells

Colon adenocarcinoma HT-29 cells (ATCC HTB-38) were employed for the adherence assays, as described by Mazariego-Espinosa et al. (2010). Clinical ETEC *lngA* positive isolates were grown in PPLO overnight at 37°C. Cell monolayers contained in 24-well polystyrene plates (Corning, NY, USA), were culture in Dulbecco's minimal Eagle medium (DMEM) (Invitrogen, Carlsbad, CA) supplemented with 10% FBS (Fetal Bovine Serum) (Gibco, USA) at 37°C under 5% CO_2_ until reaching an 80% confluence. The monolayers were infected with 10 μl of each bacterial suspension to an OD_600 nm_ of 0.5 in the McFarland scale and incubated for 4 h at 37°C and 5% CO_2_. Infected monolayers were washed three times with 1x PBS (pH 7.4) to remove unbound bacteria and incubated of 0.1% Triton-100x (Amresco Bioscience, Sweden) for 5 min; serial dilutions were plated on LB, incubated at 37°C for 24 h, and colony forming units (CFU) counted to determine CFU/ml. Adhesion assays were performed in triplicate and on three different days. Adherence values correspond to averages of triplicate assay and the corresponding standard deviation. ETEC isolates adherence was classified with cut-off CFU/ml for Enteroaggregative *E. coli* 042 is defined as two standard deviations above the mean of the positive control.

### Construction of an lngA isogenic mutant

The lambda red system was used to construct an isogenic mutant of the *lngA* gene in clinical isolate 73332 and the E9034A as described by Datsenko and Wanner (2000), Mazariego-Espinosa et al. (2010), and Mazariego-Espinosa et al. (2010). Briefly, the kanamycin resistance (*km*^*r*^) cassette from plasmid pKD4 was amplified using the following primers *lngA* F (5′ ttgagtttacctgagcagtacaggtacttagaattttgtctgcagtcaaggtgtagggctggagctgct tc 3′) and *lngA* R: (5′ tgctatccgtgtataaccggacacagaaatttaaagaagaggcaagaaaacatatgaatatcctccttag 3′). The PCR-assay was performed in a 200 μl reaction mixture containing 10 mM Tris-HCl, 3 mM MgCl_2_, 200 μM deoxynucleoside triphosphates (dNTPs), 2 U of DNA polymerase, 0.25 μM each primer, and 10 μl of template DNA. The PCR products obtained were electroporated into ETEC clinical isolate 73332 and E9034A, harboring the pKD46 plasmid, and transformants were selected for kanamycin resistance.

### Statistical analysis

The results showed are the mean of three experiments performed in triplicate on different days. Statistical analysis was done using the Student's *t*-test.

## Results

### Identification of CS21 among ETEC clinical isolates from children with diarrhea

A total of 303 clinical isolates previously identified as ETEC by serotyping, were obtained from Mexican and Bangladeshi children stool and screened by PCR for the following genes: *st*, *lt*, *lngA, cs1*, *cs3, cfa/I*, and *cofA*. Eighty one point fifty one percent (247/303) of the isolates amplified at least one gene (Table 2). The most frequent genotypes identified for the ETEC isolates were: *st*^+^/*lt*^+^ with 30.76% (76/247); *lt*^+^ 30.36% (75/247); *st*^+^ 11.33% (28/247); *lt*^+^/*lngA*^+^ 7.69% (19/247), and *st*^+^/ *lt*^+^/*lngA*^+^ with 5.26% (13/247). Differences between geographical regions were observed: in Bangladeshi isolates the *st*^+^/*lt*^+^ genotype was the most prevalent with 37% (75/201); in contrast Mexican isolates showed a prevalence of *lt*^+^ genotype of 48% (22/46) (Table 2).

**Table 2:** **Genotype detection frequency of ETEC isolates from children with diarrhea**.

**Genes**	**Percentage (positive isolates)**	**Mexican percentage (positive isolates)**	**Bangladesh percentage (positive isolates)**
*st*^+^/*lt*^+^	30.76 (76)	2.17 (1)	37.31 (75)
*lt*^+^	30.36 (75)	47.83 (22)	26.37 (53)
*st*^+^	11.33 (28)	8.70 (4)	11.94 (24)
*lt*^+^/*lngA*^+^	7.69 (19)	8.70 (4)	7.46 (15)
*st*^+^/*lt*^+^/*lngA*^+^	5.26 (13)	2.17 (1)	5.97 (12)
*lngA*^+^	2.83 (7)	10.87 (5)	0.99 (2)
*st*^+^/*lngA*^+^	2.83 (7)	2.17 (1)	2.99 (6)
*st* ^+^/*lngA*^+^/*cfaI*^+^	2.42 (6)	4.35 (2)	1.99 (4)
*st*^+^/*lt*^+^/*lngA*^+^/*cfaI*^+^	2.02 (5)	0	2.49 (5)
*st*^+^/*lt*^+^/*lngA*^+^/*cs3*^+^	1.21 (3)	6.52 (3)	0
*st*^+^/*lt*^+^/*cs3*^+^	0.8 (2)	2.17 (1)	0.50 (1)
*lt*^+^/*cs3*^+^	0.8 (2)	0	0.99 (2)
*lt*^+^/*csI*^+^	0.4 (1)	2.17 (1)	0
*st*^+^/*csI*^+^	0.4 (1)	0	0.50 (1)
*st*^+^/*lt*^+^/*lngA*^+^/*csI*^+^/*cs3*^+^	0.4 (1)	2.17 (1)	0
*lt*^+^/*lngA*^+^/*cofA*^+^	0.4 (1)	0	0.50 (1)
Total	100 (247)	99.99 (46)	100 (201)

st, gene coding for heat-stable enterotoxin; lt, gene coding for heat-labile enterotoxin; lngA, gene coding for the major subunit of longus; cfaI, gene coding for the colonization factor I; cooA, gene coding for the major subunit of CSI; cs3, gene coding for the CS3; cofA, gene coding for the major subunit of CFA/III.

### CS21 ETEC isolates carry variable enterotoxigenic virulence genes

According to Isidean et al. (2011), CS21 is perhaps one of the most prevalent CF worldwide (Isidean et al., 2011). The present study was addressed toward ETEC isolates, which possess the *lngA* gene coding for the LngA protein, the structural subunit of Longus. The results showed that the *lngA* gene was amplified in 25.10% (62/247) of the isolates. The *lngA*^+^ gene was associated to *lt*^+^ 33.87% (21/62), *st*^+^/*lt*^+^ 33.87% (21/62), *st*^+^19.35% (12/62), *cfaI*^+^17.74% (11/62), *cs3*^+^ 6.45% (4/62), *cs1*^+^ 1.61% (1/62), and *cofA*^+^ 1.61% (1/62) genes. Association of *lngA* with *lt*^+^ Bangladeshi isolates was 36% (15/42) and, just the *lngA*^+^ genotype alone with a 25% (5/20) was found in Mexican isolates. Expression of LngA in *lngA*^+^ ETEC isolates was determined by immunoblot. Fifty percent (31/62) of the isolates analyzed produced LngA, protein production distribution was similar in both geographic regions: Mexican 65% (13/20) and Bangladesh 42.9% (18/42). These data suggest that even though the isolates encode the LngA protein, specific regulatory signals may be necessary to trigger LngA expression in the remaining 50% of the clinical isolates.

### Serotyping of ETEC isolates

Thirty-one serotypes were identified in *lngA*^+^ ETEC isolates (Table S1). Three main serotypes prevailed: O128ac:H12 in 19.35% (12/62), O78:H12 and O6:H16 with 11.29% (7/62). These serotypes showed a strong association between the *lngA*^+^ gene with the *lt*^+^ and *st*^+^/*lt*^+^ genes (Table S1).

### ETEC isolates were multidrug-resistant (MDR)

**Figure 1:**
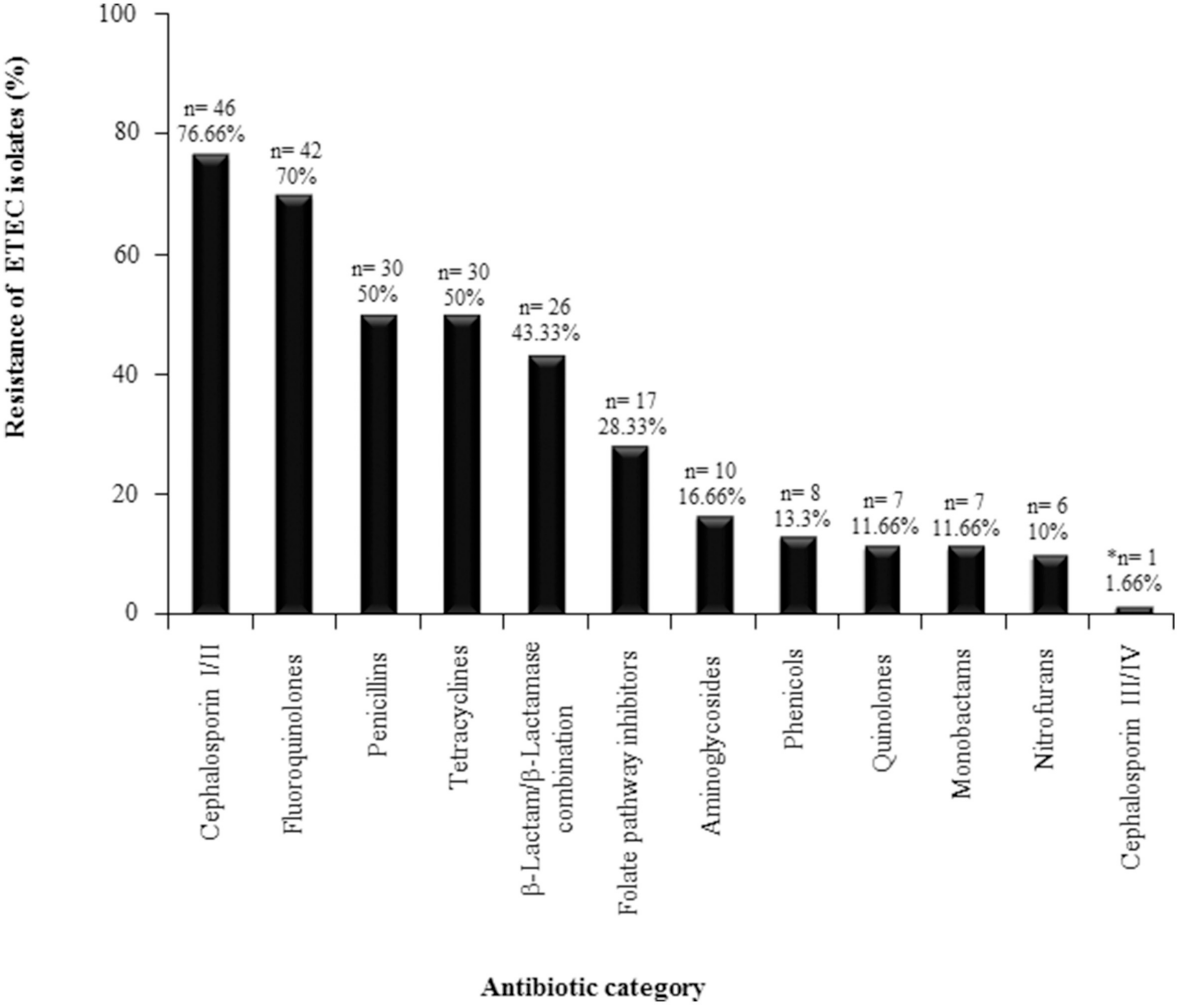
**Resistance profiles for ETEC positive isolates for the *lngA gene***. Sixty-two ETEC isolates were tested for resistance against different antibiotic categories. ^*^One ETEC isolate was resistant to a third generation cephalosporin, therefore an Extended Spectrum Beta Lactamases (ESLB) assay was performed, and was positive for this test. *n* = number of positive ETEC *lngA* isolates.

Multidrug-resistance defined as antibiotic resistance to at least three antibiotic categories is widespread among foodborne and waterborne enteric pathogens including ETEC. While ETEC treatment is not based on antibiotics in the majority of cases, evaluating antibiotic susceptibility is important in cases where antibiotic use is clinically indicated. In this study, 62 *lngA*^+^ ETEC isolates were tested for resistance to different antibiotic categories. Interestingly, 96.77% (60/62) of the isolates were resistant to at least one antibiotic category. Resistance to first and second generation cephalosporins was observed in 76.66% (46/60) of the isolates; to fluoroquinolones in 70% (42/60); to penicillin and tetracyclines in 50% (30/60); to β-Lactam/ β-lactamase inhibitor combination in 43.33% (26/60); and to folate pathway inhibitors 28.33% (17/60). ETEC antibiotic resistance to the remaining antibiotic categories was less than 17% (Figure 1). Only one ETEC isolate was resistant to a third generation cephalosporin; therefore, an Extended Spectrum Beta Lactamases (ESBL) assay was performed, and resulted positive for this test (Table S1).

**Figure 2:**
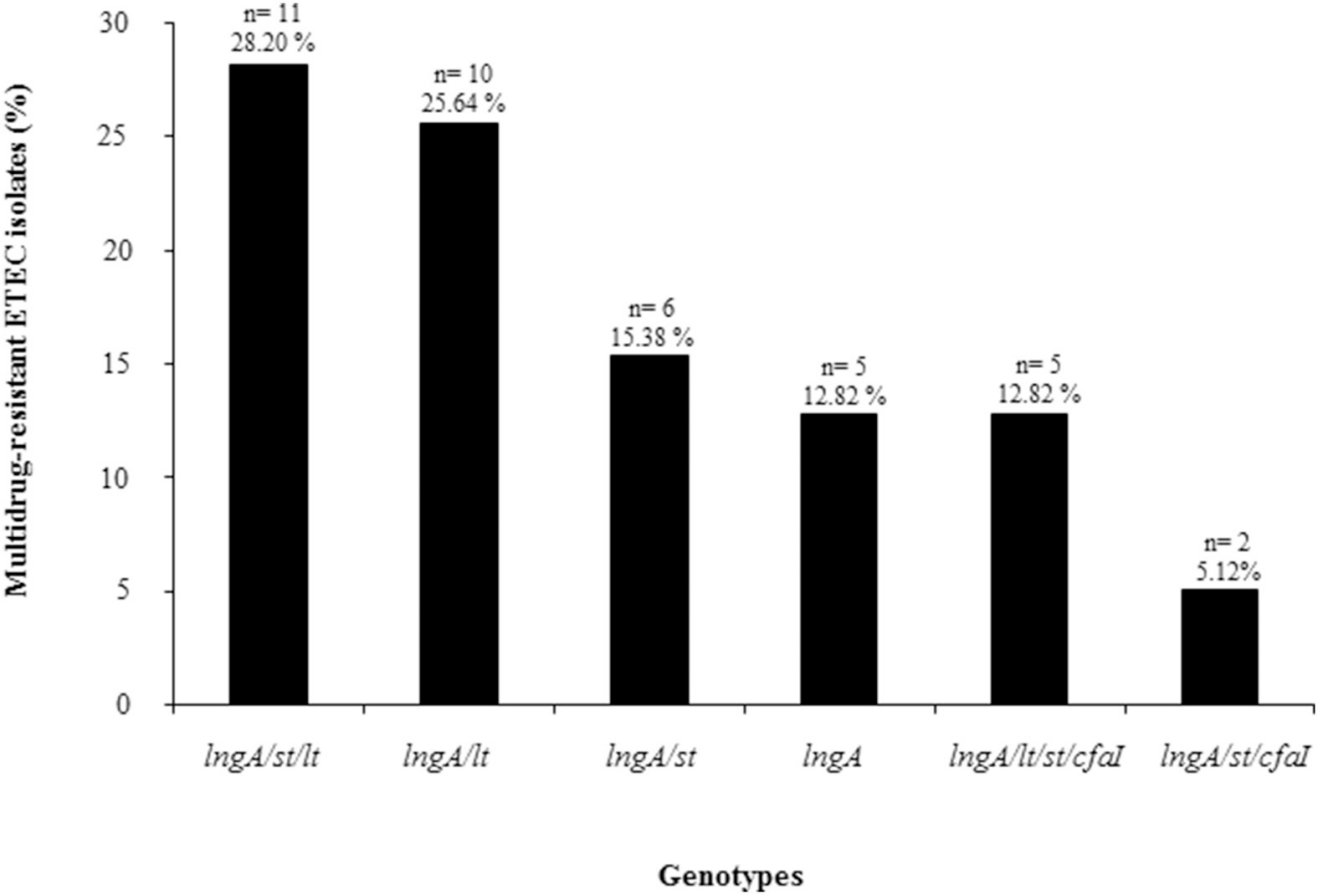
**Genotypes found in MDR ETEC isolates positive for the *lngA gene***. Sixty-five percent (39/60) of ETEC isolates were resistant to at least three antibiotic families. The association of multidrug-resistance to the genotypes was determined. *n* = number of positive ETEC *lngA* isolates.

Multidrug-resistance was identified among 65% (39/60) of the *lngA*^+^ ETEC isolates, based on geographic regions, distribution of MDR clinical isolates were from Bangladesh with 76.2% (32/42), compared with Mexican isolates with 35% (7/20) (Table S1). The association of multidrug-resistance to some genotypes was determined after observing the following distribution: 28.20% (11/39) to *lngA*^+^/*st*^+^/*lt*^+^; 25.64% (10/39) to *lngA*^+^/*lt*^+^; 15.38% (6/39) to *lngA*^+^/*st*^+^; 12.82% (5/39) to *lngA*^+^; 12.82% (5/39) to *lngA*^+^/*lt*^+^/*st*^+^/*cfaI*^+^; and 5.12% (2/39) to *lngA*^+^/*st*^+^/*cfaI*^+^ genotypes, respectively (Figure 2).

### CS21 positive ETEC isolates self-aggregate

**Figure 3:**
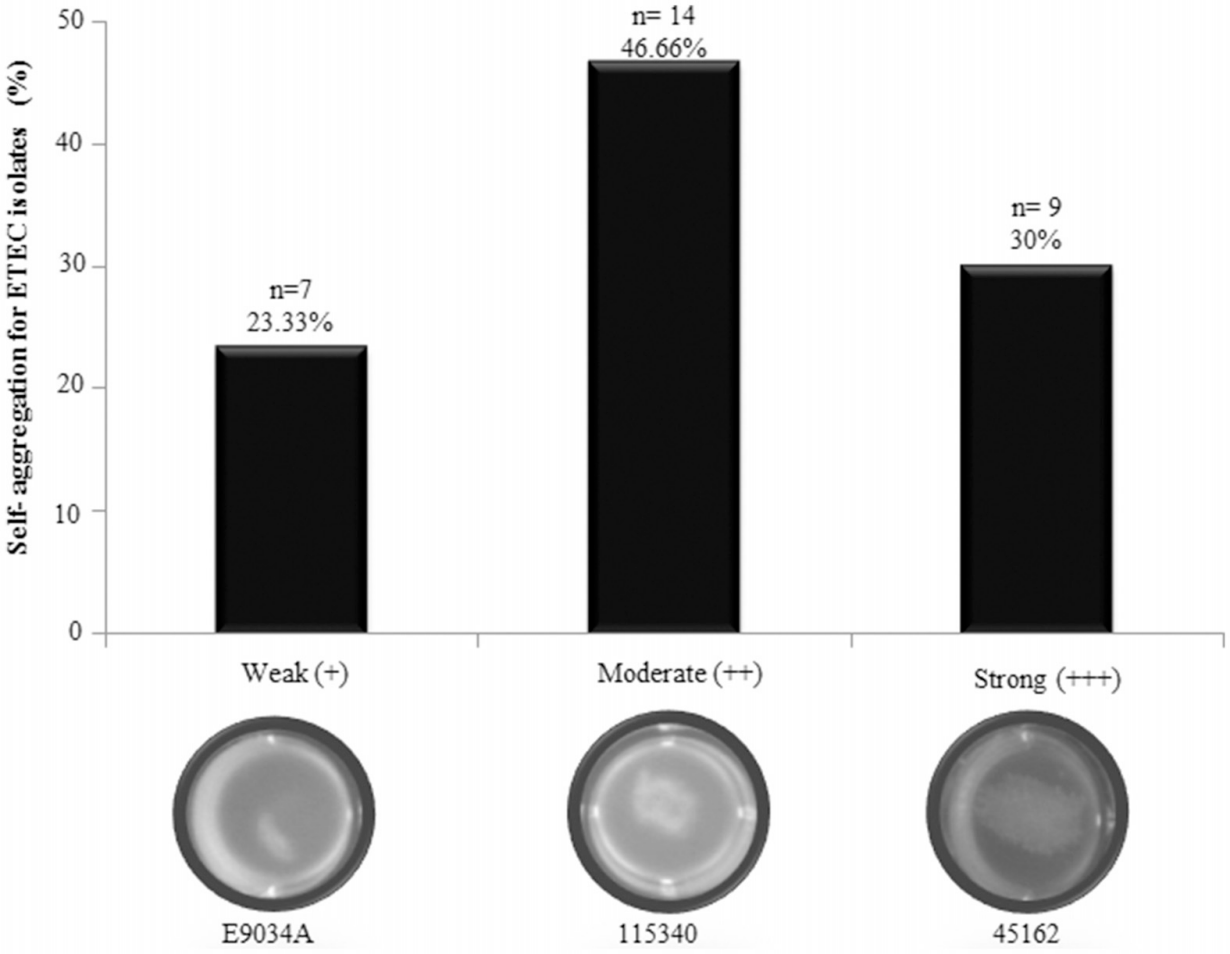
**Self-aggregation in ETEC isolates positive for the *lngA gene***. Forty-eight point eighty-three percent of ETEC isolates analyzed showed weakly (E9034A), moderately (115340), and strongly (45162) self-aggregation phenotypes. *n* = number of positive ETEC *lngA* isolates.

Self-aggregation was visualized in 48.38% (30/62) of the ETEC isolates analyzed. Self-aggregation distribution in the geographic regions was as follows: Mexican 60% (12/20) and Bangladesh 42.9% (18/42). This phenotype was classified as weak (+), moderate (++), and strong (+++) according to the levels of aggregation observed in ETEC strain E9034A, used as positive control. Figure 3 showed the self-aggregation patterns of E9034A (weak), 115340 (moderate), and 45162 (strong) ETEC isolates. Accordingly, 23.33% (7/30), 46.66% (14/30), and 30% (9/30) of the ETEC isolates showed a weakly, moderately, and strongly self-aggregation, respectively (Figure 3). Interestingly, 76.66% (23/30) of ETEC isolates expressing LngA exhibited self-aggregation, suggesting that CS21 among ETEC clinical isolates is strongly associated to this phenotype. The remaining clinical isolates 21.87% (7/32) were unable to express LngA and showed a weak or moderate self-aggregation phenotype, suggesting that CS21 contribute to the aggregate formation. To corroborate the role of *lngA* in self-aggregation, a comparison between isogenic mutants E9034A vs. E9034Δ*lngA::km* and 73332 vs. 73332Δ*lngA::km* was made, in which the loss of the phenotype was observed in both mutants (Figure S1).

### CS21 ETEC isolates form biofilm

**Figure 4:**
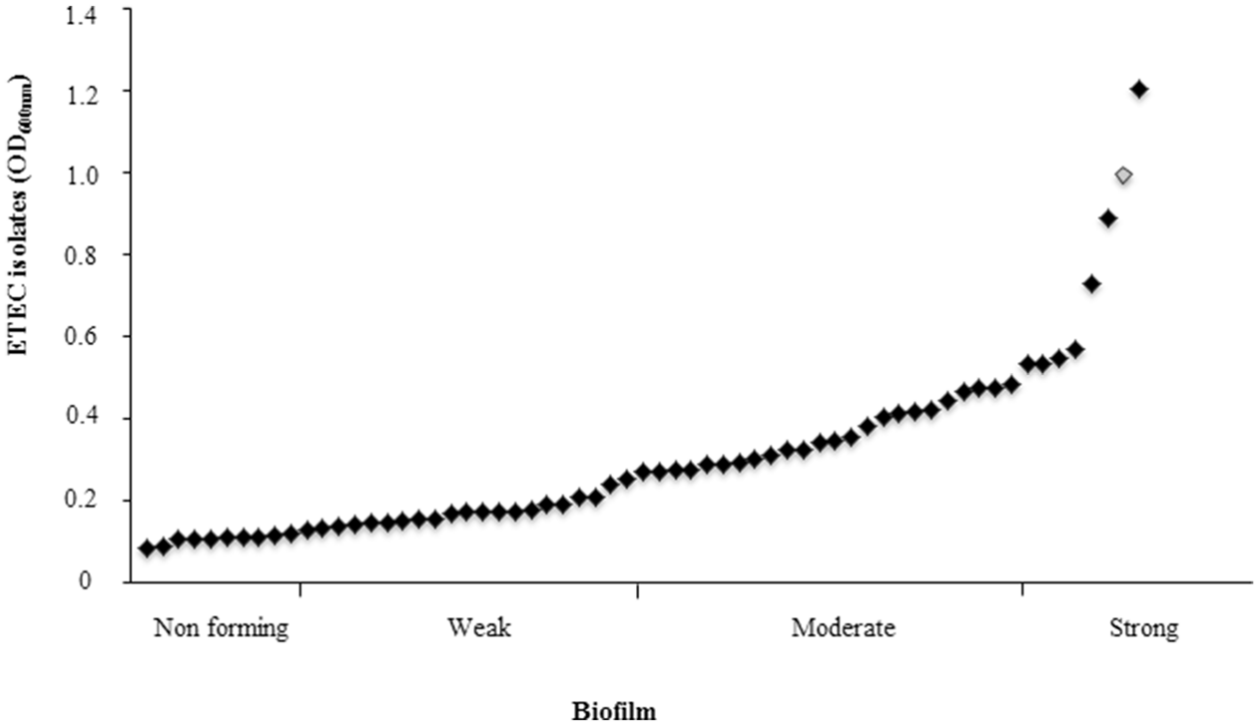
**Biofilm in ETEC isolates positive for the *lngA gene.*** From the 62 *lngA*^+^ ETEC isolates analyzed, 83.87% were able to form biofilm. Biofilm formation is critical in the pathogenesis of many human bacterial pathogens. ETEC *lngA*^−^ was used as negative control and its absorbance to classify as weakly biofilm formers (0.129–0.258); moderately biofilm formers (0.259–0.516); and strongly biofilm formers (≥0.517). Enteroaggregative *E. coli* 042 shows as rhomb on gray (positive control).

Biofilm formation is a critical event for bacterial pathogenesis. ETEC *lngA*^−^ clinical isolate 114246 was used as negative control and its absorbance was used to classify as weakly biofilm former (0.129–0.258); moderately biofilm former (0.259–0.516); and strongly biofilm former (≥0.517) (Table 3). Among *lngA*^+^ ETEC, 83.87% (52/62) formed biofilm, grouping them as: 40.38% (21/52) weakly biofilm formers; 46.15% (24/52) moderately biofilm formers, and 13.46% (7/52) strongly biofilm formers (Figure 4). Interestingly, 48.07% (25/52) of ETEC clinical isolates producing the LngA protein detected by immunoblot were associated to different levels in biofilm formation. Of those, 32% (8/25), 48% (12/25), and 20% (5/25) ETEC isolates were weakly, moderately, and strongly biofilm formers, respectively. To corroborate the role of *lngA* in biofilm formation, a comparison between isogenic mutants E9034A (0.1824 OD_600 nm_) vs. E9034Δ*lngA::km* (0.1214 OD_600 nm_) and 73332 (0.1459 OD_600 nm_) vs. 73332Δ*lngA::km* (0.1262 OD_600 nm_) was made, where the statistical analysis showed no statistical significance (*p* = 0.27) and (*p* = 0.46), respectively.

**Table 3:** **Classification of *lngA*^+^ ETEC isolates for biofilm formation**.

	***OD*_600 nm_value**	**Biofilm forming**
*OD* ≤ *OD*_c_	<0.129	Non
*OD*_c_ < *OD* ≤ 2 *OD*_c_	0.129–0.258	Weak
2 × *OD*_c_< *OD* ≤ 4 × *OD*_c_	0.259–0.516	Moderate
4 × *OD*_c_< *OD*	≥0.517	Strong

OD_c_, Optical density cut off value: average OD of negative control.

### ETEC isolates were able to strongly adhere to HT-29 cells

**Figure 5:**
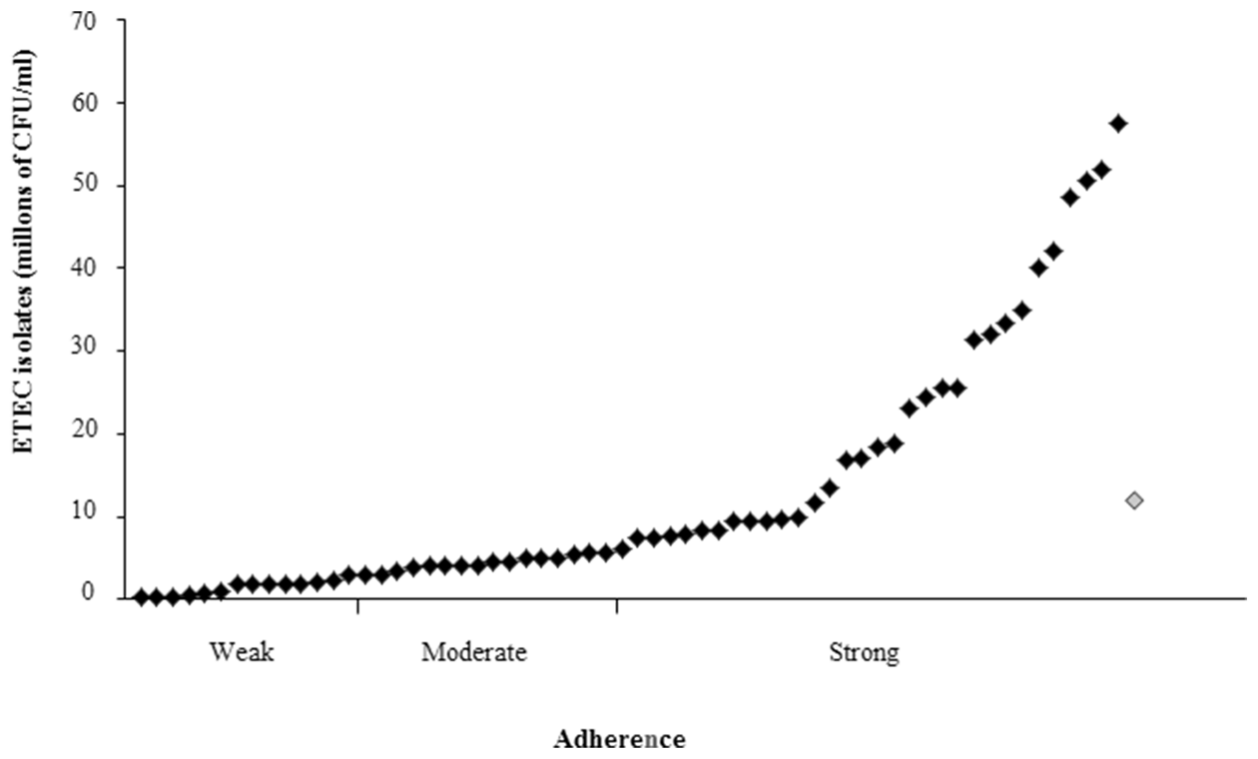
**Adherence in ETEC isolates positive for the *lngA gene***. Quantitative adherence assay determined in CFU when HT-29 cells were infected. The classification of adherence was done according to the positive control (Enteroaggregative *E*. *coli* 042) mean (11.97 × 10^6^ CFU/ml), grouped in three phenotypes: weakly (0.3–2.99 × 10^6^ CFU/ml), moderately (3.0–5.98 × 10^6^ CFU/ml), and strongly adherent (=5.99 × 10^6^ CFU/ml). Enteroaggregative *E. coli* 042 shows as rhomb on gray (positive control).

The HT-29 cells were infected with ETEC isolates positive for the *lngA* gene and the results showed different adherence values after the quantification. HT-29 cell adherence distribution was similar in both geographic regions. The adherence levels ranged from 0.3 × 10^6^ to 57.5 × 10^6^ CFU/ml (Figure 5). The classification of adherence was done according to the positive control (Enteroaggregative *E. coli* 042) mean (11.97 × 10^6^ CFU/ml), grouped in three phenotypes: weakly (0.3–2.99 × 10^6^ CFU/ml), moderately (3.0–5.98 × 10^6^ CFU/ml), and strongly adherent (≥5.99 × 10^6^ CFU/ml). Briefly, 22.58% (14/62), 25.80% (16/62), and 51.62% (32/62) of *lngA*^+^ ETEC isolates were weak, moderate, and strong adherent. The relationship between LngA expression and adherence was as follows: 29.03% (9/31) for weak and moderate adherent, and 41.94% (13/31) for strong adherent.

**Figure 6:**
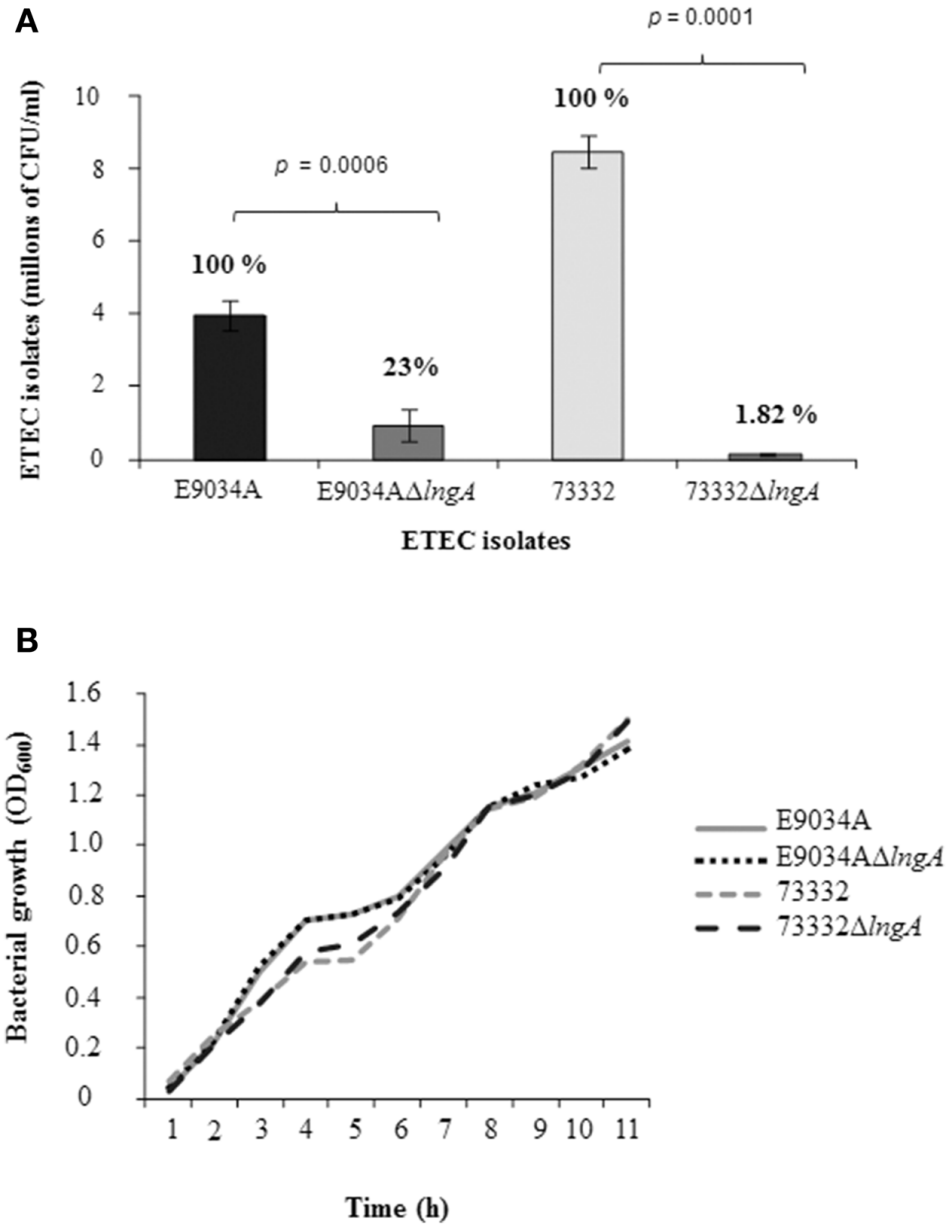
**Role of Longus in ETEC isolates E9034A and 73332. (A)** Quantitative adherence assay in CFU after 6 h of infection to HT-29 cells. The percentage of each isolate is shown in a bar graph. **(B)** Growth curve in PPLO medium to corroborate growth rate among wild type isolates and isogenic mutants in the *lngA* gene. h, hours.

Clinical isolate 73332 serotype O6:H6 carrying *lt*^+^, *st*^+^, *cs3*^+^, and *lngA*^+^ was selected to study the role of CS21 in the adherence to HT-29 cells. This isolate expresses LngA and was classified as highly adherent (8.48 × 10^6^ CFU/ml). The adherence levels were compared among ETEC wild-type (E9034A and 73332) and isogenic mutants (E9034AΔ*lngA* and 73332Δ*lngA*). Reduction of 98% (1.55 × 10^5^ CFU/ml) cell adherence with ETEC 73332 isolate (8.48 × 10^6^ CFU/ml) compared to 77% (9.33 × 10^5^ CFU/ml) reduction in ETEC E9034A strain (3.97 × 10^6^ CFU/ml) demonstrates that the reduction in the adherence was attributed to the *lngA* gene mutation (Figure 6A). A statistical difference in cell adherence reduction was observed when the *lngA* gene was deleted in ETEC clinical isolate 73332 and ETEC E9034A, with *p-*values of 0.0001 and 0.0006, respectively. A growth kinetic curve with wild type ETEC and *lngA* mutants was performed in PPLO media (Figure 6B) showing that there was no difference in growth among the wild-type and isogenic mutants ETEC isolates.

## Discussion

ETEC isolates are a frequent cause of diarrhea worldwide; ETEC causes nearly 400 million diarrhea episodes every year affecting children from developing countries as well as travelers (Wenneras and Erling, 2004; Riddle et al., 2006). ETEC colonizes the small bowel epithelium trough the expression of fimbrial and non-fimbrial CFs and producing LT and/or ST enterotoxins causing massive excretion of water and electrolytes (Qadri et al., 2005; Turner et al., 2006; Guttman and Finlay, 2008; Croxen and Finlay, 2010). After screening CFs in ETEC isolates from different geographical origins, CFA/I and Longus are the most prevalent CFs (Giron et al., 1994; Girón et al., 1995a; Gutierrez-Cazarez et al., 2000; Pichel et al., 2002). A strategy to control enterotoxigenic *E. coli* infections has been the development of a vaccine that triggers protective immunity against the most prevalent CFs (Giron et al., 1994; Girón et al., 1995a; Gutierrez-Cazarez et al., 2000; Pichel et al., 2002). In this study we analyzed a total of 303 clinical isolates obtained from Mexican and Bangladeshi children stools, classified as ETEC by serotyping. The *st*^+^/*lt*^+^ (30.76%) was the most frequent genotype amplified for ETEC isolates analyzed in this study and the frequency was higher than previously reported by Guerra et al. (2014), in which only 15% of ETEC isolates from Colombia were positive for both enterotoxin genes (Guerra et al., 2014). However, a systematic review by Isidean et al. (2011), reported a global distribution of *st*^+^/*lt*^+^ of 33% in ETEC clinical isolates, similar to the findings in this study. The frequency of the *lngA* gene among ETEC clinical isolates in several countries may vary between 8.5 and 50% (Girón et al., 1995a; Gutierrez-Cazarez et al., 2000; Nishimura et al., 2002; Pichel et al., 2002; Guerra et al., 2014). Meanwhile, in this study the *lngA* gene was amplified in 25.10% of the isolates. A lower frequency of the *lngA*^+^ gene associated to *st*^+^, *st*^+^/*lt*^+^, and *lt*^+^ genes was observed when compared with data described by Nishimura et al. (2002); which reported a frequency of 56%, 31%, and 10%, respectively (Nishimura et al., 2002). Likewise, *lngA*-positive ETEC isolates (25.10%) also harbor genes coding for other CFs. In other reports, *lngA*^+^ was most highly associated to isolates coding for CFA/II (Girón et al., 1995b; Nishimura et al., 2002) and CFA/I that has been described as the most prevalent CF in developing countries. However, in this study it appeared in low frequency 6.45%, for *cfaII* (*csI*, *cs3*), and 17.74% for *cfaI* (Pichel et al., 2002; Qadri et al., 2005). Interestingly, *lngA*^+^ isolates were found alone in 11.29% of the ETEC isolates. This result suggest that these isolates could have lost the plasmid that code for enterotoxins, leaving only the megaplasmid coding for *lngA*^+^; therefore, *lngA* is proposed as a molecular marker together with *lt* and *st* genes, for identification of the ETEC pathotype. The most prevalent serotypes found in ETEC *lngA*^+^ isolates were O128ac:H12, O78:H12, and O6:H6. The *lngA* gene was also described in a considerable number of ETEC serotypes (O2:NT, O6:NM, O6:H2, O6:H16, O6:H48, O8:H2, O8:H6, O8:H21, O8:H29, O20:H2, O25:NM, O25:H42, O49:NM, O128:NM, O139:H28, O148:H28, O153:H2, O153:H45, O158:H10, and O159:H4) (Girón et al., 1995a). These data provide further evidence of the wide distribution of CS21 among ETEC clinical isolates.

The frequency up to date reported for MDR ETEC isolates is 46.4% (Zeighami et al., 2014). In this study, 65% of ETEC isolates positive for *lngA*, were multidrug-resistant to different antibiotic categories. In particular, resistance to cephalosporins I and II (76.66%), fluoroquinolones (70%), penicillins and tetracycline (50%), β-lactam/β-lactamase combination (43.33%), folate pathway inhibitors (28.33%); and for the rest of the antibiotic categories (aminoglycosides, phenicols, quinolones, nitrofurans, monobactams, and cephalosporin III and IV) we found less than 17% of resistance. In contrast to the reports from other studies in which ampicillin, trimethoprim-sulfamethoxazole, cefazolin, and amoxicillin-clavulanate resistance was detected among 67.5%, 50%, 15%, and 5% isolates, respectively. In addition, no resistance to ceftriaxone, ceftazidime, cefepime, ciprofloxacin, and piperacillin-tazobactam was detected among ETEC isolates (Guerra et al., 2014). In other study, 47% of ETEC isolates were resistant to ampicillin, a third (37%) of the isolates was resistant to trimethoprim-sulfamethoxazole, and 24% of the isolates were tetracycline-resistant (El-Gendy et al., 2013).

Although the treatment of diarrhea caused by ETEC with antibiotic agents is generally restricted to severe and moderate hospitalized cases or immunocompromised patients, there is an increased number of clinical isolates of MDR ETEC, which could be a potential public health problem; for this reason, the study of antibiotic resistance should be carried out in combination with the frequencies of CFs and enterotoxins. We found that 46.15% of MDR ETEC isolates was able to produce the LngA protein. In this study ETEC isolates were MDR and producers of the LngA protein providing the bacteria colonization and pathogenic advantages. CS21, a type IV pilus and a common CF in ETEC, induce microcolony formation, twitching motility, adherence to cells, and self-aggregation (Clavijo et al., 2010; Mazariego-Espinosa et al., 2010). Self-aggregation has been associated to bacterial protection against antibiotics in a similar manner as bacterial biofilms avoid contact with lumen intestinal bactericidal agents such as lactoferrin, lysozyme, and secretory antibodies. In this study, 48.38% of the ETEC isolates were able to self-aggregate and 76.66% of the ETEC isolates produced the LngA protein.

Biofilms are matrix-enclosed communities of bacteria that show increased antibiotic resistance and the ability to evade the immune system (MacFarlane and Dillon, 2007). They can cause recalcitrant infections which cannot be cured with classical antibiotic therapy (Forier et al., 2014). Biofilm production and adherence mechanisms of ETEC isolates are associated with CFs and multiple genes coding for CFs located on a plasmid (CFA/I and CFA/II), as well as on its chromosome (CS2). In our study, 83.87% of the ETEC isolates tested were able to form biofilm at 24 h. Liaqat and Sakellaris (2012), described that all ETEC isolates analyzed were able to form biofilm after 60 h (Liaqat and Sakellaris, 2012). Outstandingly, most of the ETEC isolates were weak and moderate biofilm producers and a relationship was not found.

Many pathogenic bacteria have the ability to adhere to specific host tissues prior to colonization. Thus, bacterial adhesion is the initial step that precedes colonization and in some cases invasion of the epithelial surfaces (Pizarro-Cerda and Cossart, 2006). All ETEC isolates were able to adhere to HT-29 cells, and the isolates that produce the LngA protein were associated with high adherence values. Mazariego-Espinosa et al. (2010), demonstrated that CS21 produced by ETEC E9034A is involved in the phenomenon of adherence as a requirement for mediating the interaction between the bacteria and cultured epithelial cells, specifically intestinal cells (Mazariego-Espinosa et al., 2010). In this study, we demonstrate the role of CS21 for adhesion, using the E9034A prototype strain that has been widely used in other studies and a clinical isolate 73332. The aim was to analyze through quantitative studies the potential of CS21 as an adhesin in clinical isolates of ETEC during the colonization to their host.

The growth curve kinetic was performed to guarantee that the adherence phenotype was not affected by bacterial growth. Isogenic mutants in the *lngA* gene showed a reduction of adherence from 77% to 98.8%. However, Mazariego-Espinosa et al. (2010) showed a reduction of 32% in adherence levels in the E9034AΔ*lngA* when compared to ETEC E9034A (Mazariego-Espinosa et al., 2010). The difference observed in adherence levels in both mutants, could be attributed to the generation of a second mutant in the *lngA* gene employed in this study from a highly adherent colony of the E9034A wild-type strain; which, was previously selected from multiple subcultures in HT-29 cells in different days.

Antibiotic categories such as sulfonamides and beta-lactam containing compounds can be effective prophylaxis against traveler's diarrhea by deficiency of effective vaccine treatments (Brown et al., 2009) this can be explained by the different geographical origins of the Mexican and Bangladeshi isolates. However, the emergence of antibiotic resistant isolates and the genes encoding resistance proteins are known to reside on horizontal transmissible elements such as conjugative plasmids and integrons promoting the spread of resistance (Levy, 2002). The MDR ETEC isolates able to produce LngA showed features associated with self-aggregation, and adherence to intestinal cell lines. These features are important in the colonization process. Taking into consideration the frequencies of occurrence of the widely spread CFs such as CFA/I and CS21 in other geographic regions, the frequencies of occurrence of the *lngA* pilin gene found here or described by others confirm the importance of the CS21 pilus as a widely distributed antigen among ETEC isolates.

## Author contributions

Designed and conceived the experiments: Ariadnna Cruz-Córdova, Karina Espinosa-Mazariego, Sara A. Ochoa, Zeus Saldaña. Performed the experiments: Ariadnna Cruz-Córdova, Karina Espinosa-Mazariego, Zeus Saldaña, Gerardo E. Rodea, Viridiana Rodríguez-Ramírez. Analyzed the data: Ariadnna Cruz-Córdova, Karina Espinosa-Mazariego, Zeus Saldaña, Gerardo E. Rodea. Contributed reagents/materials/analysis tools: Sara A. Ochoa, Carlos A. Eslava-Campos, Firdausi Qadri, Armando Navarro-Ocaña, José Arrellano-Galindo. Reviewed the manuscript: Firdausi Qadri, Rigoberto Hernández-Castro, Oscar G. Gómez-Duarte. Wrote the manuscript Juan Xicohtencatl-Cortes.

## Ethics statement

The study was reviewed and approved by the Research (Dr. Onofre Muñoz Hernández), Ethics (Dr. Amparo Faure Fontenla), and Biosecurity (Dr. Herlinda Vera Hermosillo) Committee of Hospital Infantil de México Federico Gómez under permit numbers HIM/2014/016, HIM/2013/008, and HIM/2011/017.

### Conflict of interest statement

This work was supported by grant number CONACYT 133451 and public Federal Funds HIM/2014/016, HIM/2013/008, and HIM/2011/017 from Hospital Infantil de México Federico Gómez. The authors declare that the research was conducted in the absence of any commercial or financial relationships that could be construed as a potential conflict of interest.
