# Supplementary figures and images for: CS21 positive multidrug-resistant ETEC clinical isolates from children with diarrhea are associated with self-aggregation, and adherence
Source: Front Microbiol. 2014 Dec 17;5:709. doi: 10.3389/fmicb.2014.00709 (PMC4297921; doi:10.3389/fmicb.2014.00709)

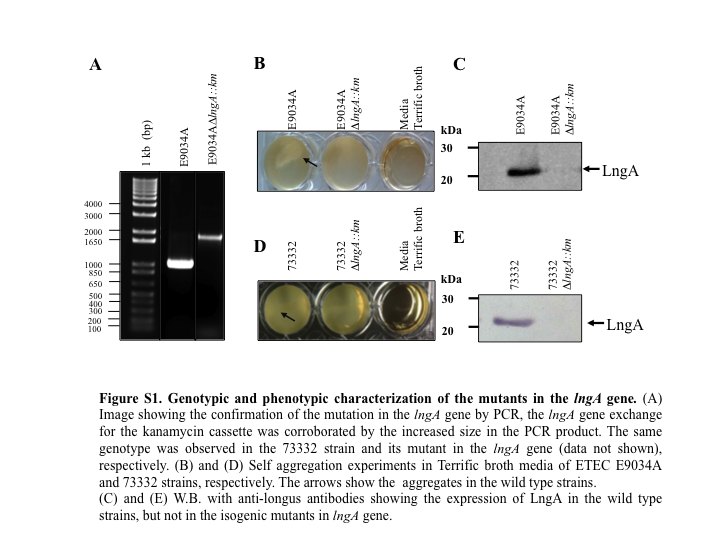

Supplement: Supplementary file 2 [file Image1.TIFF]
